# Wearable devices and cardiovascular health: revolutionizing remote monitoring and disease prevention

**DOI:** 10.1093/eurheartj/ehag189

**Published:** 2026-03-25

**Authors:** Andrew M Hughes, Daniel J Taylor, Paul D Morris, Evan L Brittain

**Affiliations:** Division of Cardiovascular Medicine, University of Minnesota, Minneapolis, MN, USA; Division of Clinical Medicine, School of Medicine and Population Health, University of Sheffield, Sheffield, UK; Insigneo Institute for in Silico Medicine, University of Sheffield, Sheffield, UK; Division of Clinical Medicine, School of Medicine and Population Health, University of Sheffield, Sheffield, UK; Insigneo Institute for in Silico Medicine, University of Sheffield, Sheffield, UK; Department of Cardiology, South Yorkshire Cardiothoracic Centre, Sheffield Teaching Hospitals NHS Foundation Trust, Sheffield, UK; Division of Cardiovascular Medicine, Vanderbilt University Medical Center, 2525 West End Avenue, Office 680, Nashville, TN 37203, USA

**Keywords:** Wearable devices, Behavioural monitoring, Cardiovascular risk, Cardiovascular disease, Artificial intelligence

## Abstract

Wearable devices are transforming cardiovascular medicine by enabling continuous monitoring of physiologic and behavioural measures outside of traditional clinical settings. Smartwatches and activity trackers, the most widely used wearables, employ motion and biometric sensors to measure physical activity, sleep quality, heart rate, and rhythm. By converting health goals into objective, quantifiable measures, wearable devices empower patients to assume a more active role in their health while providing clinicians with novel opportunities for longitudinal, real-world assessment. Clinical applications span the cardiovascular continuum from lifestyle interventions targeting physical activity and sleep to the remote management of chronic conditions such as heart failure. Widespread clinical adoption of wearables remains limited by challenges, such as variability in device methodology, data outputs, validation, and intended use; incompatibility with existing electronic health records; and the lack of standardized, evidence-based workflows for clinicians to efficiently interpret and act upon wearable data. This review summarizes the current landscape of wearable technologies in cardiovascular medicine by highlighting key clinical applications, evidence gaps in the existing literature, the role of artificial intelligence, and barriers to implementation. We discuss strategies to enhance clinical integration and strengthen the current evidence base while also providing practical guidance to help clinicians navigate commonly encountered clinical scenarios.

## Graphical Abstract

**Graphical Abstract:**
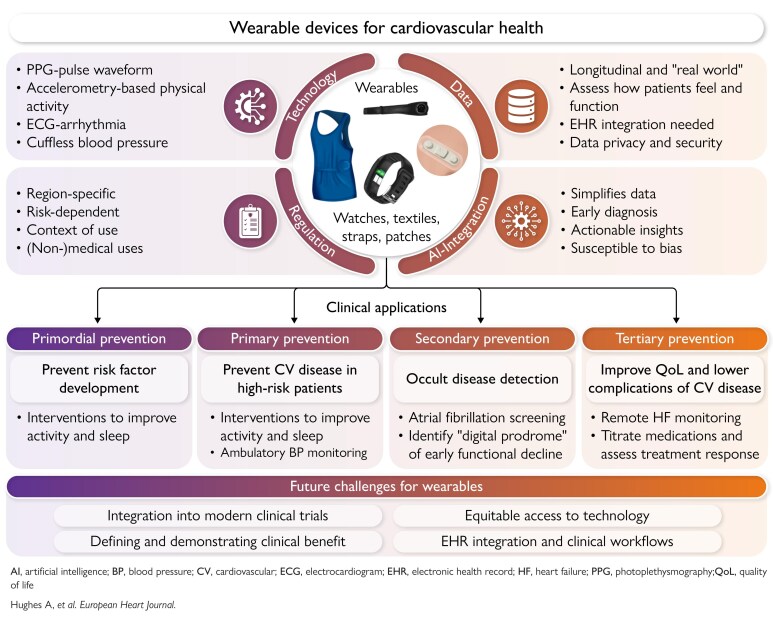

Wearable devices leverage a range of sensor technologies to capture longitudinal, real-world physiologic data with expanding clinical applications across the cardiovascular disease spectrum, though their impact remains limited by several ongoing challenges.

## Introduction

Wearable devices, such as smartwatches and activity trackers, are popular with patients because they are user-friendly and provide real-time health insights. Wearable technologies rely on motion and biometric sensors to capture physiologic and behavioural measures, such as physical activity (PA), sleep, heart rate (HR), heart rate variability (HRV), heart rhythm, blood pressure (BP), and BP variability.^1^ By transforming abstract health goals into measurable data that patients can actively track, wearables empower patients to assume a more active role in their health.^2^ Wearables may shift patients from passive recipients of sporadic, clinic-based assessments to active participants with continuous monitoring of key health metrics in their “real-world” environment. Wearables are also ushering in a paradigm shift for clinicians who are learning to interpret data from devices they may not have prescribed.

Although clinical integration of wearable data remains in its infancy, these devices hold considerable promise to advance personalized care. Potential applications include lifestyle interventions for primary prevention, disease screening of high-risk individuals, and remote management of patients with established cardiovascular conditions. However, current clinical infrastructure cannot support widespread wearable device integration. Essential next steps include integration of wearable data into the electronic health record (EHR) and the development of automated alerts that clinicians can efficiently interpret. While prior research has highlighted the exciting potential of wearables in cardiovascular medicine, future studies must address unresolved challenges, including data harmonization across devices, recruitment and retention of diverse populations, and alignment of wearable-derived metrics with established clinical endpoints.

This review article builds on prior work^3–7^ by summarizing the current state of wearables in cardiovascular medicine. We offer a forward-looking perspective that provides practical guidance for clinicians encountering wearables in practice and outline strategies for researchers and key stakeholders to enhance study design, promote equitable use of artificial intelligence (AI) tools, and facilitate integration of these devices into healthcare systems.

## Overview of wearable technologies

**Figure 1:**
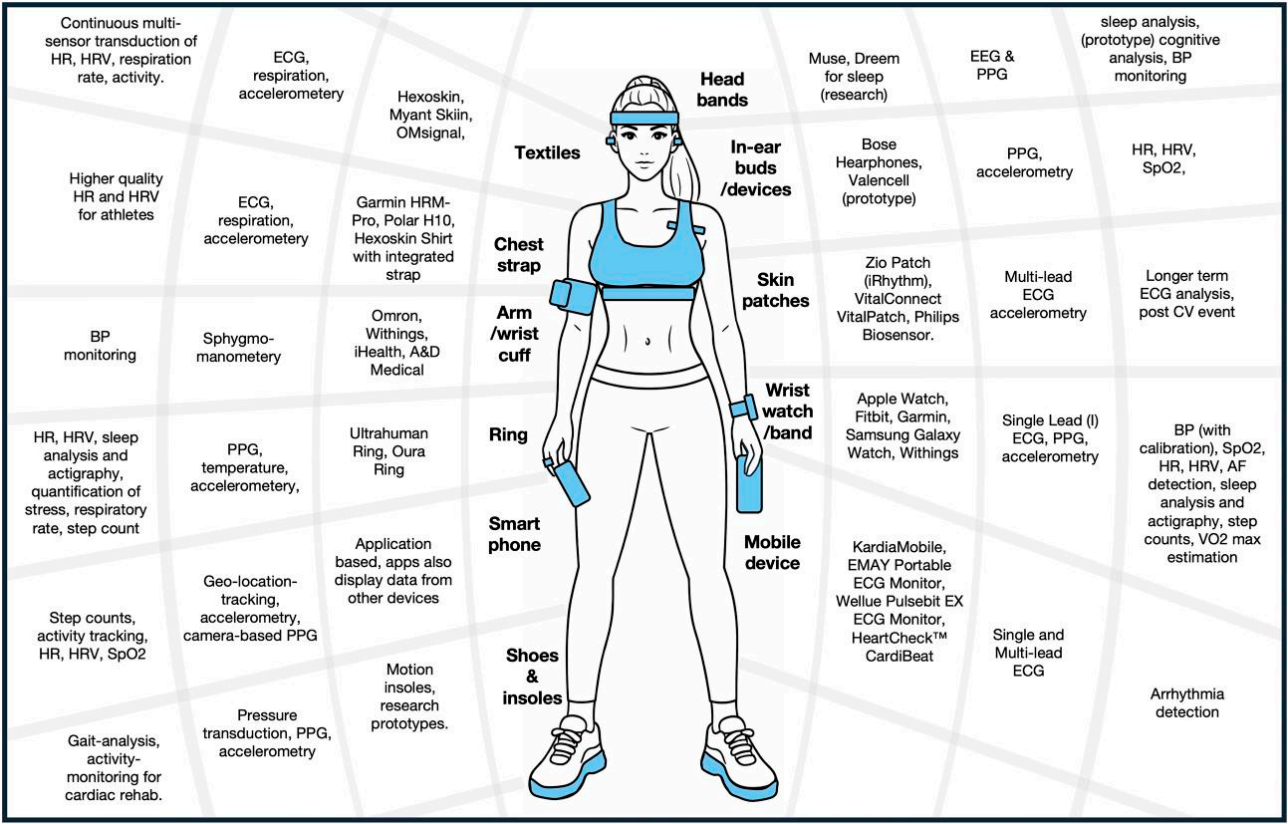
Examples of common wearable and portable cardiovascular devices (in blue) in various apparel. Examples are labelled (outwards) with examples of manufacturers and models, the types of transducer technologies used, and the type of ‘health’ data generated. HR, heart rate; HRV, heart rate variability; ECG, electrocardiogram; BP, blood pressure; PPG, photoplethysmography; SpO_2_, oxygen saturation; AF, atrial fibrillation; VO_2_ max, maximal oxygen consumption; CV, cardiovascular; EEG, electroencephalogram

Several key sensor technologies capture cardiovascular data, and wearables come in diverse forms, such as wristbands, smartwatches, and sensor-embedded apparel (*Figure 1*).

*Electrocardiography (ECG)* measures the voltage difference between two points. In wrist-worn smartwatches, ECG recordings are user initiated, often prompted by abnormal photoplethysmography (PPG) signals or symptoms. Electrocardiography tracings are obtained using one sensor within the wristwatch and another sensor elsewhere on the device, which is contacted by the opposite hand. This produces a tracing analogous to Lead I of a standard 12-lead ECG.^8^ Handheld devices, such as KardiaMobile, generate single- or six-lead ECG tracings when fingertips are placed on the electrodes of the device. Algorithmic analysis of these recordings aids in detecting arrhythmias.^9^ Unlike on-demand recordings, devices such as Holter monitors and external loop recorders track ECG tracings continuously over periods ranging from 24 h to several weeks.^10^

*Accelerometry* technology has advanced considerably with smaller devices providing increasingly accurate transduction of motion (in x, y, and z axes), and gyroscope technology captures rotational motion to determine orientation (e.g. posture). Accelerometry is incorporated into lifestyle devices to derive PA metrics (e.g. step count and activity intensity) and frailty assessment (e.g. fall detection and gait analysis). When combined with PPG data, it can be used to deduce sleep–wake patterns and differentiate rapid eye movement (REM) and deep slow-wave phases of sleep.^11^ It can also be used to correct for motion artefacts in other signals like PPG.

*Photoplethysmography* utilizes a light source shone into the skin. The proportion of absorbed, transmitted, and reflected light depends on its wavelength and the local blood volume, which fluctuates with pulsatile blood flow. Photo-sensitive transducers detect transmitted or reflected light, from which a pulse waveform can be reconstructed and a range of data derived, including HR, HRV, and oxygen saturation (SpO_2_). Initial atrial fibrillation (AF) screening trials relied on PPG-based irregular pulse notifications. Additionally, there is strong concordance between PPG-derived HRV and ECG for low-frequency parameters, but the concordance is only moderate for high-frequency measures.^12^ Artificial intelligence and wearable device technology have improved HRV measurement, which is a promising research tool for CV risk stratification, though clinical utility remains limited by inconsistent data acquisition and analysis methods.^13^Combined with multi-site sensing or, more commonly, ECG sensing, additional metrics can be derived. For example, the pulse transit time (time from the ECG R-wave to the PPG-sensed pulse ‘arrival’ time) correlates with systemic BP, as does the pulse wave velocity.

*Cuffless BP* technologies predominantly rely on pulse waveform analysis and pulse transit time and require calibration against conventional upper-arm cuff devices to ensure measurement accuracy.^14^ The degree to which cuffless BP readings depend on the initial calibration, and whether these devices can reliably track BP trends over time, remains uncertain.^15^ Furthermore, measurements are susceptible to individual factors, such as body posture, ambient temperature, and vascular tone.^14^ Although several cuffless BP devices are commercially available and BP variability is emerging as an important indicator of impaired cardiovascular regulation with prognostic significance, their routine clinical use is not currently recommended due to ongoing concerns regarding the calibration process and user-dependent factors.^14,16^

*Temperature* measurements commonly rely on contact-based thermistors or infrared sensors. To minimize motion artefacts and environmental confounders, wearable devices typically track temperature trends during sleep, when activity and ambient variability are reduced. While many wearable devices capture temperature data, the clinical significance of temperature in cardiovascular disease remains poorly defined.^3,17^

*Other sensors* Some sensor technologies remain predominantly in the research or medical device space rather than consumer wearable devices. Phonocardiography (PCG) is a mechano-acoustic sensing technique that records the mechanical vibrations produced by cardiac activity, such as valve closure and blood flow turbulence, to capture the timing, intensity, and characteristics of heart sounds and murmurs.^18,19^ Phonocardiography signals are typically acquired using contact microphones or piezoelectric sensors, and novel wearables are working to integrate ECG and PCG for advanced cardiovascular monitoring.^20^ Ballistocardiography (BCG) uses accelerometry to non-invasively detect mechanical forces caused by each heartbeat to estimate HR, HRV, stroke volume, cardiac output, arrhythmia detection, and sleep analysis.^21^ Remote dielectric sensing utilizes the dielectric properties of tissues to detect transmitted or reflected low-power electromagnetic signals to estimate tissue fluid content, which has shown benefit for monitoring pulmonary congestion in heart failure (HF).^22,23^

## Regulation and classification of wearable devices

Wearable devices are characterized as consumer-grade (lifestyle) devices or medical devices based on the nature of regulatory approval. In Europe, regulation is overseen by the European Commission. A certified medical device must receive a Certificate Europe (CE) mark under the Medical Device Regulation by an accredited Notified Body according to risk:

**I. Low-risk devices** are non-invasive/minimal contact (e.g. non-invasive monitoring devices). Mostly self-certified, Notified Bodies are involved for measurement accuracy.

**IIa. Low–moderate risk devices** are short-term invasive diagnostic or therapeutic devices. Notified Body reviews technical documentation and clinical evaluation is required.

**IIb. Moderate–high risk devices** include long-term invasive devices and those administering or controlling therapy. Examples include angioplasty balloons and intra-aortic balloon pumps. It requires deeper Notified Body scrutiny and robust clinical data.

**III. Highest-risk devices** include implantable, life-sustaining devices within the central circulation (e.g. coronary stents, prosthetic valves, and pacemakers). They require full Notified Body review with expert panel scrutiny and mandatory post-market clinical follow-up.

In the USA, medical devices are regulated by the Food and Drug Administration (FDA) Center for Devices and Radiological Health. Food and Drug Administration classification describes the regulatory burden, not just risk. The recently updated European regulations have narrowed the gap between FDA and CE pathways. Unlike in Europe, where (decentralized) Notified Bodies assess devices, the FDA is responsible for its own assessment:

- **Low-risk devices**: General controls (e.g. manufacturer registration, device listing, quality system regulation, and adverse event reporting) are usually sufficient. Around 90% are exempt from pre-market submission, and minimal clinical evidence is required.
- **Moderate risk:** Combination of general and specific controls. The latter involve FDA device-specific guidance, post-market surveillance, clinical registry or study data, details of contraindications, procedural instructions, and safety testing (e.g. biocompatibility, electrical safety, sterilization, and validation).
- **High risk:** Life-sustaining, implantable, and life-supporting devices that are high risk if device failure occurs. Require rigorous pre-market approval with extensive bench and animal testing, prospective clinical trials, and detailed manufacturing inspection. Examples include coronary stents and pacemakers.

Some consumer lifestyle devices are also approved as medical devices, but it is important to understand what each device feature is approved for according to the manufacturers’ *indications for use* statement. Examples include the Apple Heart Watch (Series 4 onwards), Samsung Galaxy Watch (Active2, Watch3, and later), FitBit Sense (Charge 5 onwards), Omron HeartGuide, and the Withings ScanWatch (see Supplementary data online, Table S1, for regulatory approval details).

## Indications for use

Even for approved medical devices, users must strictly follow manufacturer instructions for use. For example, the Apple Heart Watch has US FDA (Class II) and CE mark approval as a medical device to determine the presence of AF, sinus rhythm, and high HR on a classifiable waveform. However, the *indications for use* statement^24^ clarifies a number of important caveats:

- Unsuitable for AF detection at HR between 100 and 150 b.p.m.
- Not recommended for users with other known arrhythmias.
- Electrocardiography data are intended for informational use only, and users should not interpret or take clinical action based on the device output without consultation with a qualified healthcare professional.
- Electrocardiography waveform is meant to supplement rhythm classification for the purposes of discriminating AF from sinus rhythm and is not intended to replace traditional methods of diagnosis or treatment.
- Electrocardiography app is not intended for use by people under 22 years old.

Given the increasing number of shop-bought devices, all with their own technologies, measurements, device-specific approvals, and indications for use statements, the appropriate interpretation of this data will be challenging for many physicians. For a more detailed review of device development, regulation, validation, and interpretation, please see the recent clinical consensus statement from the ESC Regulatory Affairs Committee.^25^

### Validation

All devices need to prove safety and reliability. Beyond this, *clinical* validation may involve comparison of a device-derived measurement against the gold standard. This is relatively simple in the context of established measurements, such as BP and HR, but more novel measurements, such as activity and sleep quality, are harder to validate against a gold standard. Regulatory validation requirements depend on device type (lifestyle vs medical) and risk classification, with high-risk medical devices requiring the most stringent data. Validation studies do not need to prove efficacy and do not necessarily require a randomized controlled trial (RCT). However, they do need to demonstrate safety in the target population, as well as reliability, and justify what is described in the indications for use statement.

## Clinical applications of wearable devices

**Figure 2:**
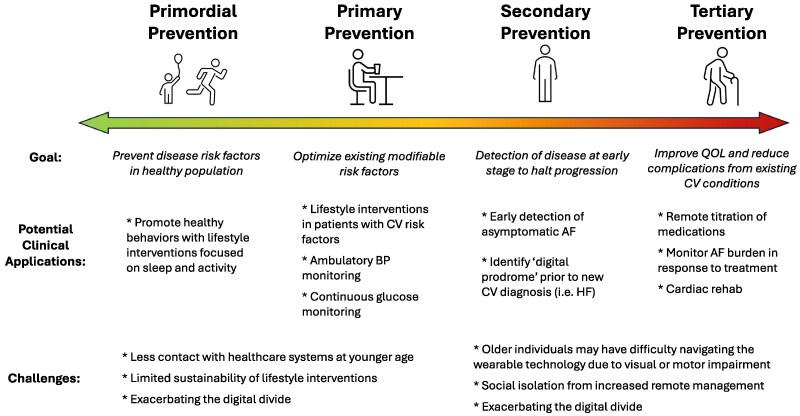
Clinical applications of wearable devices across the entire lifespan. AF, atrial fibrillation; BP, blood pressure; CV, cardiovascular; HF, heart failure; QoL, quality of life

**Figure 3:**
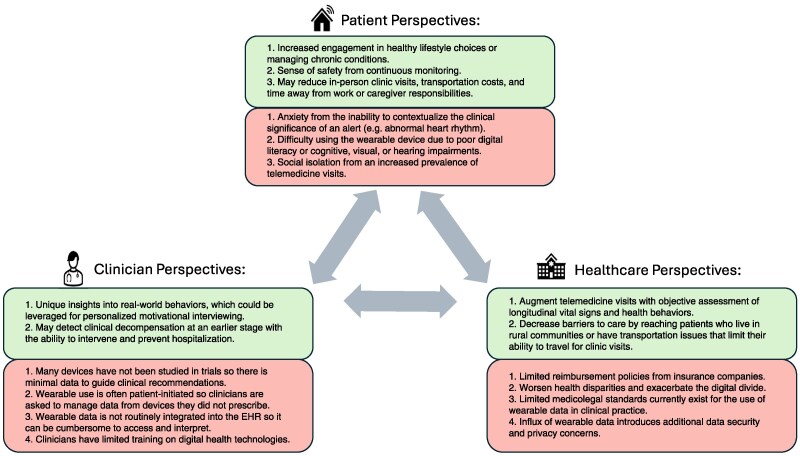
Perspectives from key stakeholders on wearable devices in clinical practice: the potential benefits are included in the top text box, and the unresolved challenges that limit widespread adoption of wearable devices are highlighted in the lower text box. EHR, electronic health record

Studies based on wearable technologies utilize consumer devices for (i) long-term, passive, observational monitoring, (ii) as part of an interventional tool, or (iii) assessment of a therapeutic response. While this section is not a systematic review, the included studies (*Table 1*) highlight pivotal clinical trials and novel proof-of-concept studies across the spectrum of disease (*Figure 2*). The insights gained, along with perspectives of key stakeholders (*Figure 3*), may shape future research and integration into clinical practice.

**Table 1:** Selected clinical studies with wearable devices

Study	Design and participants	Wearable device	Measurement	Key findings
Primordial prevention				
Au *et al.*^26^	SR/MA of 21 RCTs *N* = 3676 children and adolescents	Variety of wearable activity trackers	Daily step count and activity intensity	Activity trackers increased daily steps (SMD .37; *P* = .013) but not MVPA compared with controls
Tang *et al.*^27^	SR/MA of 12 RCTs *N* = 1693 healthy adults	Variety of wearable activity trackers	Daily step count and activity intensity	Wearable-based interventions achieved a modest, short-term improvement in activity (SMD .449; *P* = .01)
Primary prevention				
Hodkinson *et al.*^28^	SR/MA of 38 RCTs *N* = 4203 adults with cardiometabolic conditions	Variety of wearable activity trackers	Daily step count and activity intensity	Wearable-based interventions significantly increased PA levels (SMD .72; *P* < .001), especially when the intervention included consultation with a healthcare professional
ENGAGE Study^29^	RCT *N* = 500 adults from low SES with ASCVD condition or ASCVD risk **≥** 7.5%	Fitbit Alta or Fitbit Inspire (Fitbit, Inc.)	Daily step count and activity intensity	An intervention with self-selected goals and immediate implementation (compared with assigned goals with gradual implementation) significantly improved activity by 1384 steps/day and 4.1 min of MVPA, which were sustained at 8 weeks
NAVIGATOR Study^30^	RCT *N* = 9306 adults with impaired glucose tolerance and an additional CV risk factor	Pedometer (Accusplit)	Daily step count	Baseline step count (HR per 2000 steps/day .90, 95% CI .84–.96) and change in step count over 12 months (HR .92; 95% CI .86–.99) were inversely associated with CV risk
Patel *et al.*^31^	RCT *N* = 361 adults with uncontrolled Type 2 diabetes	Withings Activite Steel (Withings, Inc.)	Daily step count	Gamification interventions that promoted competition or support increased daily steps by 606 and 503, respectively, over 1 year
Secondary prevention				
Apple Heart Study^32^	Prospective, multicentre study *N* = 419297 adults without AF	Apple watch (Apple, Inc.)	Irregular heart rhythm (PPG)	.52% of participants had an irregular heart rhythm notification with a PPV of 84% for AF detection
Fitbit Heart Study^33^	Prospective, remote clinical trial *N* = 455 699 adults without AF	Various Fitbit devices (Fitbit, Inc.)	Irregular heart rhythm (PPG)	1% of participants had an irregular heart rhythm notification with a PPV of 98% for AF detection
AMALFI Study^34^	RCT *N* = 5040 adults **≥** 65 years old with moderate–high stroke risk in the UK	14-day wearable ECG patch (Zio XT, iRhythm)	ECG	Compared with usual care, AF screening with an ECG patch had a modest increase in AF diagnosis at 2.5 years (ratio of proportions = 1.26, 95% CI 1.02–1.57, *P* = .03), but no significant difference in the stroke rate (rate ratio, 1.08, 95% CI .76–1.53)
Tertiary prevention				
RATE AF Trial^35^	RCT *N* = 53 adults with permanent AF and HF	Fitbit Charge 2 (Fitbit, Inc.)	Heart rate and daily step count	Digoxin and beta-blockers had similar effects on heart rate at rest and with exertion, highlighting the feasibility of wearables to monitor treatment response and support clinical research
CHIEF-HF Study^36,37^	Remote and decentralized RCT *N* = 476 adults with HF irrespective of EF or diabetes status	Fitbit Versa 2 (Fitbit, Inc.)	Daily step count	Daily step count was associated with KCCQ quality of life scores at the time of enrolment and in response to canagliflozin treatment with evidence that a 1000 step increase is clinically meaningful
LINK HF Study^38^	Prospective, multicentre study *N* = 100 adults with HF, NYHA Class II–IV, and recent HF hospitalization	Multisensor wearable chest patch (Vital Connect)	PA, ECG, skin impedance, temperature	ML algorithms detected HF readmission with 85% specificity and 76%–88% sensitivity, which is similar to implanted devices. Alerts preceded the readmission by 6.5 days
Maddison *et al.*^39^	Non-inferiority RCT *N* = 162 patients with coronary heart disease	BioHarness 3 chest worn sensor (Zephyr Technology)	Heart rate, respiratory rate, single-lead ECG and PA	Compared with traditional centre-based CR programmes, the remote exercise-based CR program was non-inferior for functional capacity (similar VO_2_ max at 12 weeks), and it had lower programme delivery costs
Nagatomi *et al.*^40^	RCT *N* = 30 outpatients with HF and physical frailty	Fitbit Inspire (Fitbit, Inc.)	Daily step count	A home-based CR programme facilitated by a consumer-wearable device and smartphone app improved 6MWD by more than 50 m and improved muscle strength in a safe manner

ASCVD, atherosclerotic cardiovascular disease; AF, atrial fibrillation; CR, cardiac rehabilitation; CV, cardiovascular; EF, ejection fraction; ECG, electrocardiogram; HR, hazard ratio; HF, heart failure; KCCQ, Kansas City Cardiomyopathy Questionnaire; ML, machine learning; VO_2_, maximal oxygen consumption; MA, meta-analysis; MVPA, moderate-to-vigorous physical activity; NYHA, New York Heart Association; PPG, photoplethysmography; PA, physical activity; PPV, positive predictive value; RCT, randomized controlled trial; SES, socioeconomic status; SMD, standardized mean difference; SR, systematic review; 6MWD, 6-min walk distance.

### Primordial prevention

Primordial prevention aims to mitigate disease risk factor development in a healthy population by addressing underlying health behaviours and environmental determinants.^41^ Meta-analyses suggest that activity trackers may lead to short-term, transient increases in daily step counts in children/adolescents^26^ and healthy adults.^27^ The interventions in these studies included variable access to health-based applications, feedback, and counselling.

Multifaceted interventions that incorporate behavioural change techniques appear more effective compared with passive interventions.^42^ Strategies such as individualized feedback with personalized text messages,^43^ adaptive goal setting,^44^ or gamification^45,46^ have been leveraged to enhance engagement with mixed results. For example, the BE FIT trial used a gamified design in which family members were grouped into teams. Each day, one member was randomly selected to represent the team. If that individual failed to achieve their step goal from the previous day, the entire team lost points. This approach leveraged principles of competition psychology, including loss aversion and variable reinforcement, to enhance motivation and promote sustained behavioural change.^45^ Despite employing evidence-based strategies, the BE FIT trial, like many others, struggled to sustain the increased activity levels. These findings underscore the notion that wearable devices, while useful facilitators, are not sufficient as standalone tools to drive lasting improvements in activity. Rather, they must be integrated alongside established methods, such as lifestyle counselling, to address motivation, accountability, and habit formation.^47^ Defining an optimal multi-dimensional approach should be a priority of future research.

### Primary prevention

Primary prevention focuses on reducing the risk of clinical cardiovascular events by targeting established, modifiable risk factors, such as hypertension and diabetes.

Most existing wearable-based studies focused on PA, but incorporating sleep may enhance risk stratification and primary prevention efforts.^48^

Studies consistently associate lower PA levels with an increased risk of adverse cardiovascular outcomes.^30,49–51^ Certain populations, such as individuals from lower socioeconomic backgrounds, are underrepresented in existing device-based PA studies.^52^ Individuals from lower socioeconomic backgrounds may require tailored interventions to ensure equitable distribution of resources^29^ and tackle the high prevalence of cardiac comorbidities within this demographic. In the ENGAGE study, a wearable-based intervention with immediate implementation of self-selected goals (rather than gradual introduction of pre-assigned goals) significantly improved activity levels amongst economically disadvantaged adults with atherosclerotic risk factors.^29^

### Secondary prevention

Secondary prevention emphasizes detection of diseases in the latent or early phase to halt disease progression.^53^ Population-wide screening programmes are often limited by time or cost considerations. The remote nature of wearable devices has the potential to implement efficient and economically viable alternatives for large-scale screening efforts.

Atrial fibrillation is particularly attractive for early detection with wearable devices; it is often asymptomatic, and stroke prevention strategies are well established. The three largest trials of routine AF screening in the general population recruited over 1 million participants^32,33,54^ and relied on PPG-based irregular pulse notifications. Collectively, these studies demonstrated the feasibility of screening initiatives to detect AF; however, diagnostic accuracy was moderate, and the implications of population-wide AF screening for hard clinical endpoints remain unclear. The ongoing Heartline study will evaluate whether population-wide AF screening with an Apple watch improves time to diagnosis and cardiovascular outcomes.^55^

Wearable devices may also detect a ‘digital prodrome’ prior to clinically significant events, such as a new diagnosis of HF. In a high-risk cohort, Fitbit-derived activity levels decreased significantly in the months preceding a new diagnosis of HF compared with matched controls.^56^ By detecting changes in digital biomarkers, wearables may identify functional decline and prompt expedited clinical evaluation to potentially facilitate intervention and ameliorate disease progression.

### Tertiary prevention

Tertiary prevention aims to reduce complications and improve quality of life (QoL) in patients with established disease. Several studies investigated whether remote monitoring interventions with wearable devices decrease HF readmissions or mortality, but the results were mixed.^57–61^ Interventions were typically multifaceted, incorporating ECG devices, BP monitors, scales, and increased clinician follow-up. Consequently, isolating the independent effect of the wearable device is challenging. Although increased provider contact and close follow-up may account for some observed effects, this should not be viewed solely as a confounding artefact, as it may represent an important mechanistic mediator through which remote monitoring interventions improve outcomes. Wearable-based surveillance has the potential to provide incremental benefit beyond clinician contact by enabling earlier detection of physiologic deterioration and clinical decompensation.

Guideline-directed medical therapy (GDMT) remains underutilized in chronic HF.^62–64^ Digital interventions with wearable devices may help facilitate the initiation or up-titration of these medications. The AIM-POWER trial^65^ is randomizing participants with symptomatic HF on suboptimal GDMT to remote management with BioVitalsHF (Biofourmis, Boston, MA) or usual care. BioVitalsHF is an AI-driven decision-support platform that integrates ambulatory physiologic data (e.g. HR, BP, and body weight) to provide clinicians with actionable recommendations for medication titration. The BioVitalsHF platform provided titration recommendations every 2 weeks, but clinicians had the final decision regarding medication changes.

The completely remote, decentralized CHIEF-HF trial evaluated canagliflozin vs placebo in patients with HF. The trial found that canagliflozin safely improved QoL and symptom burden at 12 weeks, but did not increase total daily steps as measured with a Fitbit Versa 2.^36^ Similarly, the TRACE study used wrist-worn actigraphy and found that selexipag treatment did not improve real-world activity levels in patients with pulmonary arterial hypertension.^66^ These findings highlight that while pharmacologic interventions may yield net patient benefit, the relationship between effective pharmacotherapy and PA is complex and influenced by human behaviour, motivation, and other contextual factors. However, the ability of wearables to objectively assess treatment response throughout follow-up is a key strength which will, when used appropriately, improve understanding of the relationship between pharmacotherapy and PA in modern clinical trials.

Beyond HF, consumer wearables show promise for monitoring arrhythmia burden, as demonstrated in the RATE AF trial, where they revealed that digoxin and beta-blockers had similar effects on HR at rest and with exertion. This highlights the potential for wearable devices to facilitate dynamic outpatient arrhythmia monitoring.^35^ Lastly, wearable devices also have an important role in delivering remote cardiac telerehabilitation programming^67^ and potentially sustaining activity levels after rehabilitation programme completion.^68^

## Advancing study design with wearable devices

**Figure 4:**
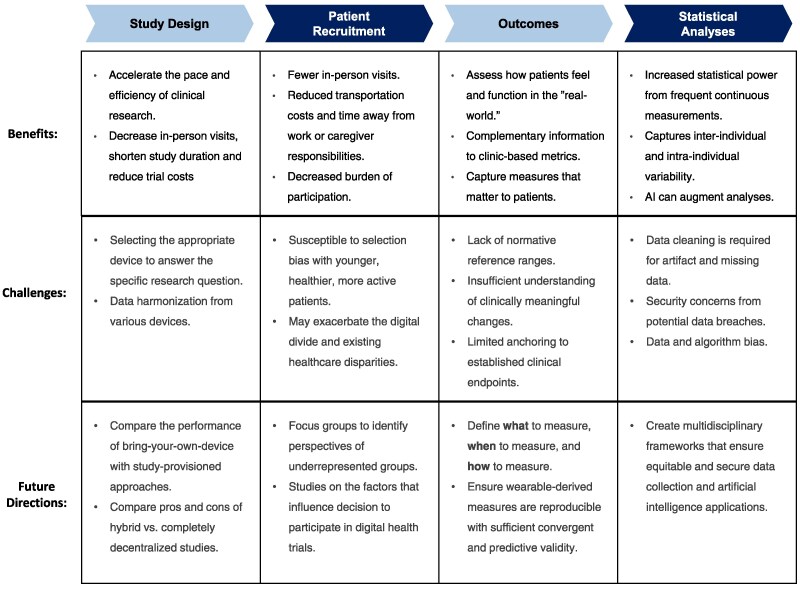
Wearable devices in the design of clinical studies: This figure highlights key considerations for the different aspects of clinical study methodology that wearable devices have the potential to impact. AI, artificial intelligence

**Figure 5:**
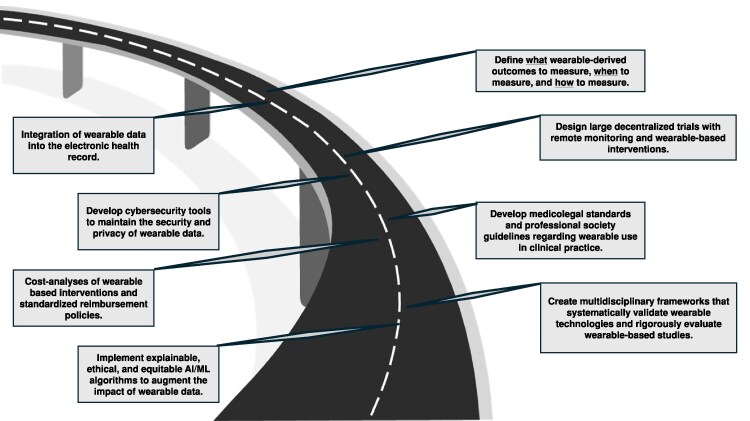
A road map to improve the usage of wearable devices in clinical practice. AI, artificial intelligence; ML, machine learning

Wearable devices capture real-world, free-living data outside of traditional clinical settings to generate rich datasets. These technologies offer significant potential to improve clinical studies, but several challenges must be addressed for wearable devices to reach their full potential in clinical research (*Figure 4*). In this section, we explore how wearable devices can enhance study methodology and outline a road map of potential solutions to overcome unresolved challenges (*Figure 5*).

### Study design

#### Benefits

Wearable devices can accelerate the efficiency and pace of clinical research through decentralization in which study procedures and data collection happen outside of a clinical setting (e.g. a participant’s home).^69^ The high-frequency, continuous data collection from wearable devices increases the data density and statistical power compared with sporadic clinic-based assessments. As an illustrative example, a decentralized hypertension trial could incorporate ambulatory BP monitoring using wearable cuffless devices to examine the impact of frequent, longitudinal measurements. High-density BP data collected in participants’ usual environments may increase data granularity, improve statistical power, facilitate earlier detection of treatment effects, and reduce white coat-related measurement bias. Access to more frequent BP measurements could support protocol-guided adjustments of antihypertensive therapy by providing more timely information on treatment response. Additionally, remote BP monitoring may reduce reliance on in-person visits, thereby decreasing participant burden and potentially lowering trial costs.

#### Challenges

Existing wearable-based studies have employed a diverse array of devices and interventions, yielding heterogeneous results. Researchers must understand the intended use and the inherent limitations of each device to appropriately select and tailor the technology to address the specific research question. Clinical studies may provide participants with the same device (study-provisioned) or use data from a participants’ personal device [bring-your-own device (BYOD)].^70^ Study-provisioned devices minimize variability by using a single device, but it is associated with increased study costs.^70^ The BYOD approach may exacerbate existing health disparities associated with technology access or digital literacy. Additionally, the vast number of commercial devices makes data harmonization difficult as activity measures are captured differently across various devices. For example, a “step” measured by a Fitbit device is not exactly the same as a “step” measured by an Apple watch.

#### Potential solutions

To mitigate challenges with data harmonization, studies may utilize a single device model for an individual’s entire monitoring period. This approach preserves internal validity, identifies intraindividual changes, and supports accurate longitudinal analyses. However, new models or software updates may compromise researchers’ ability to maintain device consistency, especially in studies with long-term follow-up. Developing proactive, prespecified change-control plans can help navigate unforeseen changes in device availability or functionality.^71^

### Patient recruitment

#### Benefits

Wearable devices collect outcomes remotely and reduce the number of in-person study visits, transportation costs, and time away from work or caregiver responsibilities.^72–74^ Clinical studies that decrease the participation burden achieve more efficient recruitment and study enrolment.^75,76^ For example, compared with DAPA-HF,^77^ the DAPA-MI^78^ trial reduced in-person study visits by 60%, and participants reported an improved patient experience.^72^

#### Challenges

While wearables can reduce study visits and improve patient adherence, these benefits may be offset by reduced data accuracy or variability in self-monitoring, such as inconsistent device use or improper placement. This highlights the importance of validation studies, standardized protocols, careful data cleaning, and participant training, which are discussed throughout the manuscript. Digital health studies are also susceptible to selection bias and may overrepresent younger, healthier, more active, or financially affluent individuals with increased access and familiarity with wearable technologies.^79^ Although ownership remains lower compared with younger generations, many (33%) adults over the age of 75 own a wearable device, and the vast majority of older adults (90%) are willing to share wearable data with a provider.^80^ Older adults may face barriers navigating wearable technologies due to visual, hearing, and motor difficulties.^81^ Lastly, populations in resource-limited settings, including low-income countries or rural communities, experience disparities in internet connectivity, smartphone access, EHR infrastructure, and availability of trained personnel. These infrastructure and resource gaps contribute to a digital divide in which access to digital health tools is unevenly distributed across and within countries, thereby highlighting an evidence gap and equity concern in digital medicine that warrants focused attention.^82^

#### Potential solutions

Healthcare providers and researchers must collaborate with government agencies, technology companies, and local communities to effectively address the digital divide, especially in low-resource settings. Such partnerships are essential to prioritize investment in infrastructure development, digital literacy initiatives, and human capacity building.^82,83^ Studies should also investigate the factors that influence an individual’s decision to participate in digital health trials so that recruitment and retention can be tailored appropriately to avoid unintended selection bias.^70^ At this stage, researchers should feel empowered to enrol large cohorts while ensuring that additional support is available for participants who encounter difficulties navigating wearable technology.

### Outcomes

#### Benefits

Wearables are uniquely equipped to capture how patients feel and function in their daily lives. Wearables continuously monitor physiologic and behavioural data, such as activity, sleep patterns, and HR, in an objective manner that is free of recall bias. Longitudinal monitoring mitigates the temporary influences of clinic-based assessments (e.g. 6-min walk test and polysomnography), which are susceptible to how a patient feels at the moment of testing. Wearables provide complementary information to clinic-based outcome measures. Functional and symptomatic assessments may capture patient’s goals more effectively than traditional morbidity/mortality measures. For example, a study of aortic valve interventions demonstrated that nearly half of the individuals pursued intervention to maintain functional status and participate in a hobby compared with 7% of patients with mortality as their primary motivation.^84^ The real-world data captured by wearables in future studies may allow clinicians to pursue more personalized risk/benefit assessments and provide recommendations that align with patient expectations.^70,85^

#### Challenges

There remains a lack of published reference ranges for wearable-derived measures. Establishing normative values can be challenging due to the heterogeneity amongst consumer wearable devices, and existing datasets may be biased with fewer individuals from underrepresented groups.^86^ Studies can still focus on changes over time rather than absolute benchmarks.^86,87^ However, there is an insufficient understanding of what constitutes a clinically meaningful change for wearable-derived measures,^88^ and a limited number of wearable-derived measures have been anchored to established clinical endpoints. The CHIEF-HF trial shows that wearable-derived activity measures were associated with QoL scores at the time of enrolment and longitudinally in response to canagliflozin treatment,^37^ but more studies are needed to establish the relationship between wearable measures and traditional clinical endpoints.

#### Potential solutions

The HFC-ARC working group is a multidisciplinary partnership to improve functional and symptomatic endpoints in HF clinical trials.^88^ HFC-ARC outline five ‘expectations’ for actigraphy to be considered a valid functional endpoint. Similar criteria could be applied to consumer grade wearables. The five criteria are content validity (measures the concept of interest), convergent validity (correlation with an established functional/symptomatic endpoint), predictive validity (association with future endpoints like hospitalization or mortality), reproducibility, and responsiveness to change (detects differences over time).^88^

### Data analysis

#### Benefits

Longitudinal monitoring captures both interindividual and intraindividual variability.^89–92^ This is especially beneficial in heterogeneous populations, as it can detect unique individual-level changes and facilitate responder analyses.^89^ Longitudinal monitoring generates more frequent measurements, which may add statistical power at a reduced sample size, making trials more time- and cost-efficient.^89,93^ Finally, AI algorithms can augment the data captured by wearables and potentially usher in a new era of cardiovascular health monitoring and predictive analytics.^94,95^

#### Challenges

Wearables generate large, raw datasets with inherent artefacts and missing data (e.g. when the device is not worn). This requires thoughtful curation to generate an accurate dataset amenable to statistical analyses and AI model training.^95,96^ Wearable devices transfer data via Bluetooth and Wi-Fi, thereby introducing security risks for data breaches or unauthorized access to the datasets.^97^ Artificial intelligence introduces additional security concerns as it may be possible to re-identify individuals following unauthorized access to a training database.^94,98^

#### Potential solutions

To address concerns about data privacy, bias, and transparency in AI-driven applications, we need frameworks that align with existing legal standards, such as the UK Data Protection Act and European Union (EU) AI Act.^95^ The UK Data Protection Act classifies sensitive health data collected by wearables as special category personal data, requiring adherence to principles of minimization (collect only what is necessary), purpose limitation (use data only as intended), and security measures. Patients retain rights over their data, and research use typically involves pseudonymization and ethical oversight. The EU AI Act establishes a risk-based regulatory framework, imposing stricter requirements on high-risk applications, including healthcare, with requirements for data quality, transparency, human oversight, and accountability.

The ESC Regulatory Affairs Committee established a Task Force to review existing assessment frameworks and the evidence supporting mobile health solutions. In this context, they highlight the CEN-ISO/TS 82304-2 quality assessment framework for health and wellness apps. Manufacturers complete an 81-item assessment that produces a quality label showing the app’s main benefit, whether approval from a healthcare professional is required, and colour-coded scores for four quality indicators: (i) *healthy and safe*, (ii) *easy to use*, (iii) *secure data*, and (iv) *robust build*. While the framework is designed for apps, similar approaches could be applied to provide transparent, evidence-based guidance about wearable devices.^25^

## Wearables in the clinical workflow

Despite the widespread adoption of wearables by consumers,^99,100^ integration into the EHR and clinical workflows remains limited. Recent data from the *All of Us* research programme highlights the potential clinical value of linking wearables with EHR data.^101–105^ For example, long-term step count and sleep quality measures were strongly associated with future risk of numerous chronic diseases.^101,102,104^ These data also suggest the potential for individualizing activity recommendations to reduce chronic disease risk (e.g. obesity).^101,103^ However, in current practice, wearable data exists in digital silos, accessible to patients via vendor apps but rarely structured or standardized in a way that facilitates clinical decision-making. This disconnect limits the clinical utility of wearables and may frustrate patients and clinicians.

Several factors hinder the integration of wearable data into EHR systems. First, the lack of interoperability across devices and platforms is an important barrier. Some wearable manufacturers use proprietary data formats or application programming interfaces (APIs), which complicates integration into an EHR. Even when integration is technically feasible, data are often unstructured, voluminous, and not clearly differentiated from other sources in the EHR (e.g. information collected in clinic). This makes it difficult for clinicians to identify relevant trends, validate measurements, or reconcile discrepancies between patient-reported and device-collected data.

Second, workflow disruption poses a practical challenge. Clinicians are already overburdened with information and notifications. Additional streams of patient-generated data without guidance on clinical thresholds and appropriate action may frustrate clinicians. At scale, these data could increase documentation burden, unnecessary testing, and unreimbursed time if not presented in a clinically digestible format.

The goal of integration efforts should be to present clinicians with actionable data in an easily interpretable fashion. Evidence-based, automated decision support is currently lacking, but a pragmatic approach is human-in-the-loop models where allied health professionals (e.g. digital health coaches, nurses, and exercise physiologists) review and respond to worrisome trends. Despite the challenges limiting wearable use in clinical practice, providers are often asked to interpret the patient-generated data. *Table 2* provides commonly encountered scenarios and reasonable advice on how to navigate these conversations based on the available evidence.

**Table 2:** Common pitfalls and advice for frequently encountered clinical scenarios with wearable devices

Scenario	Common pitfall	Advice
Patient shows smartwatch alert for “possible atrial fibrillation”	Equating alert with diagnosis	Confirm with clinical-grade ECG; assess stroke risk and symptom correlation
Elevated nighttime HR on wearable	Overinterpretation as pathology	Consider artefact, stress, or autonomic dysfunction; assess for trends over days/weeks
Patient uses multiple wearables (e.g. Oura and Apple Watch)	Data overload, conflicting metrics	Focus on one validated device; prioritize reproducibility and trends over precision and outliers
‘Low SpO₂’ alert during sleep	Misinterpretation leading to unnecessary referrals	Confirm with formal testing if indicated; review in context of device limitations and symptoms
Request to adjust medications based on wearable BP	Acting on unvalidated measurements	Recommend validated home BP cuffs; avoid medication changes based solely on wearable data
Concern over low activity alerts post-hospitalization	Premature judgement of recovery	Reassure and monitor trends in context of expected recovery; integrate with rehab or recovery plans
Patient uses wearables to justify stopping medications	Selective interpretation of ‘normal’ data	Educate on limits of device accuracy; emphasize evidence-based risk assessment
Abnormal HRV prompts anxiety	Misunderstanding of normative variation	Normalize fluctuations; clarify that HRV is not yet a diagnostic tool; identify possible explanatory factors (e.g. alcohol, medications, and poor sleep)

BP, blood pressure; ECG, electrocardiogram; HR, heart rate; HRV, heart rate variability; SpO_2_, oxygen saturation

Efforts to improve integration are underway. Electronic health record vendors such as Epic and Cerner offer modules that allow wearable data to populate in patient charts or patient portals through APIs, such as Apple HealthKit and Google Fit. Some health systems are experimenting with data triage algorithms or dashboards to flag deviations from individual baselines rather than absolute thresholds. These early implementation efforts emphasize the importance of contextualizing data such that patterns are prioritized rather than individual data points.

## Artificial intelligence and wearables

The fusion of wearables and AI represents a major inflection point in CV health monitoring. Wearables generate continuous, high-frequency physiologic data for which traditional analytic approaches are not well suited. Artificial intelligence and machine learning algorithms may be leveraged to extract signal from noise, identify novel phenotypes, and generate actionable insights from complex data streams.

Several AI-enabled applications have reached clinical relevance. Deep learning models trained on single-lead smartwatch ECGs demonstrated high accuracy in detecting AF,^106–108^ left ventricular systolic dysfunction,^109,110^ and QT prolongation.^111–113^ These models leverage subtle temporal and morphologic features not visible to the naked eye. Beyond diagnosis, AI is being tested to track trajectories, detect decompensation, and personalize risk stratification.^114–117^ In the future, algorithms could identify subtle changes in sleep regularity or circadian rhythm disruption that precede clinical deterioration.^118^ Combined with demographic, genomic, or environmental data, wearable-derived features may improve risk prediction in a manner that is dynamic and individualized.

Several challenges must be addressed before these tools are routinely deployed in clinical practice. Many models are trained on homogeneous datasets that underrepresent older adults, racial and ethnic minorities, and individuals with lower digital literacy. This raises the risk of algorithmic bias, where AI systems underperform or misclassify in subgroups. There is also concern of overfitting to device-specific idiosyncrasies, meaning that an algorithm developed for one brand of wearable may not generalize to others. Furthermore, most commercially available algorithms are “black box” models with limited transparency, hindering clinician trust and regulatory acceptance. The integration of explainable AI methods can demystify algorithmic decision-making by highlighting the physiological features driving a prediction. This may reassure clinicians that models rely on biologically plausible signals rather than data artefacts. Such transparency is also a growing regulatory imperative for agencies, such as the FDA, and frameworks, such as the EU AI Act, that increasingly view interpretability as a prerequisite for safety and accountability in medical devices.

To mitigate these risks, future development should prioritize explainability, open-source validation, and external calibration across diverse populations and devices. One such approach is federated learning, which allows models to be trained on decentralized data without moving the data itself. Moreover, integration with clinical endpoints and adjudicated outcomes will be essential to transition from pattern recognition to clinical utility.

## Conclusions

Wearable devices are poised to revolutionize personalized cardiovascular medicine through continuous real-world monitoring. By enabling long-term monitoring, early detection, and timely intervention, wearables may improve clinical management across the disease spectrum, support lifestyle behaviour change, and empower patients to take an active role in their health. Integration into the EHR, combined with AI-driven analytics, can convert the rich data streams into reliable, actionable insights that may improve patient outcomes. Wearables support decentralized trials with longitudinal monitoring of novel outcome measures that capture how patients feel and function. Challenges remain, including data variability, the need for validation across devices and populations, integration into clinical workflows, and ensuring equitable access. Future research should prioritize validation studies, outcome-driven trials, implementation strategies, and clinician training to ensure wearable technologies become scalable, clinically meaningful tools that advance precision cardiovascular care.

## Supplementary Material

ehag189_Supplementary_Data

## Declarations

### Data Availability

No data were generated or analysed for or in support of this paper.
