# Supplementary material for: Wearable devices and cardiovascular health: revolutionizing remote monitoring and disease prevention
Source: Eur Heart J. 2026 Mar 25;47(18):2130–45. doi: 10.1093/eurheartj/ehag189 (PMC13178683; doi:10.1093/eurheartj/ehag189)
Supplement: ehag189_Supplementary_Data [file ehag189_supplementary_data.docx]

**Supplementary Table 1.** FDA and EU MDR classification for some common (lifestyle) cardiovascular devices and applications.

| **Device** | **Features and functions** | **FDA** | **EU MDR** |
| --- | --- | --- | --- |
| **Apple Watch (ECG app, Series 4+ and Ultra 1-3)** | Single-lead ECG, AF detection  Other wellness features not regulated as medical device | Class II | Class IIa |
| **Samsung Galaxy Watch Active2, Watch3, Watch4/5+ (ECG, AF detection)** | Single-lead ECG, AF detection + hypertension (requires calibration, limited regions only)  Other wellness features not regulated as medical device | Class II | Class IIa |
| **Withings ScanWatch** | Single-lead ECG, AF detection + SpO2 pulse oximetry  Other wellness features not regulated as medical device | Class II | Class IIa |
| **Google Pixel watch 3+, Fitbit Sense / Charge 5 (similar sensor technology)** | Single lead ECG, AF and Loss of Pulse Detection (calls emergency services)  Other wellness features not regulated as medical device | Class II | Class IIa |
| **AliveCor KardiaMobile**  **(mobile but not ‘wearable’)** | Single-lead ECG, AF detection. 6 and 12-lead versions have extended functionality including QTc and MI detection. | Class II | Class IIa |
| **Selected Garmin watches** | Single lead ECG, AF detection.  Other wellness features not regulated as medical device | Class II | Class IIa |
| **Omron HeartGuide** | Wearable BP monitor. Blood pressure (oscillometric) and heart rate measurement. | Class II | Class IIa |
| **Huawei’s Watch D2, 5 and GT 5 Pro** | Single-lead ECG, AF detection + BP measurement.  Europe only.  Other wellness features not regulated as medical device | - | Class IIa |
| **Oura, Whoop, Motiv, Amazon Halo, Happy, Viatom, Ultrahuman, RingConn, Rings** | Wellness features only. Not regulated as medical device. | - | - |
| **Movano Evie Ring** | SpO2 pulse oximetry and heart rate  Other wellness features not regulated as medical device | Class II  (some restrictions) |  |

FDA; US Food and Drug Administration, EU MDR; European Union Medical Devices Regulation, AF; atrial fibrillation. Most devices’ ECG feature is FDA approved via the 510(k) pathway which requires manufacturers to demonstrate the device’s *substantial equivalence* to an existing, approved device. Always check the manufacturers’ indications /instructions for use statement for each product; devices are not stand-alone *diagnostic* devices. This is a guide only and not an exhaustive list of applications and metrics measured and reported. Not all models carry all features and many approvals and classifications are territory specific. Usually, it is a specific function that is approved, rather than the device itself.

**Further information**

1. <https://regulatoryinfo.apple.com/regulatorydata>
2. <https://www.samsung.com/uk/support/regulatory-information/>
3. <https://support.withings.com/hc/en-us/articles/360015551577-ScanWatch-Regulatory-statement>
4. <https://www.withings.com/eu/en/compliance?srsltid=AfmBOooStSfdOcoUkXy2PMaT4nQC_yYaVlJX40-GQix4-a6yIFNQDQ9Z>
5. <https://support.google.com/product-documentation/answer/9204905>
6. <https://www.fitbit.com/sg/legal/safety-instructions>
7. <https://alivecor.zendesk.com/hc/en-us/articles/1500000462022-User-Manuals>
8. <https://support.garmin.com/en-GB/ql/?focus=manuals>
9. <https://www.accessdata.fda.gov/cdrh_docs/pdf18/k182166.pdf#:~:text=INDICATIONS%20FOR%20USE%20%5B807.92(a)(5)%5D%20The%20device%20is,and%20gives%20a%20warning%20signal%20with%20readings>.
10. <https://omronhealthcare.com/storage/pdfs/heartguide-wearable-blood-pressure-monitor-bp8000-l-im-en_2872436-0c.pdf>
11. <https://consumer.huawei.com/en/support/wearables/>
12. <https://www.accessdata.fda.gov/cdrh_docs/pdf24/K241090.pdf>
13. <https://support.eviering.com/hc/en-us/articles/17552933642381-Product-Safety-Use>
